# Negative effects of high public debt on health systems facing pandemic crisis: Lessons from COVID-19 in Europe to prepare for future emergencies

**DOI:** 10.3934/publichealth.2024024

**Published:** 2024-04-11

**Authors:** Mario Coccia, Igor Benati

**Affiliations:** CNR – National Research Council of Italy, Department of Social Science and Humanities, IRCRES, Torino, Italy

**Keywords:** COVID-19, government debt, crisis management, preparedness, systemic vulnerability, public health, public health governance, pandemic crisis, Europe

## Abstract

The investigation goal here was to analyze how the level of public debt affects preparedness of health systems to face emergencies. In particular, this study examined the negative effects of high public debt on health systems of European countries in the presence of the COVID-19 pandemic crisis. Empirical evidence revealed that European countries with a lower level of government debt as a percentage of GDP both in 2009 and 2019 (the period before the arrival of the pandemic) had lower COVID-19 fatality rates compared to countries with higher levels of public debt. The explanation is that high levels of public debt in countries trigger budget constraints that limit their ability to allocate resources to healthcare systems (e.g., health expenditures and investments), weakening health system performance and causing systemic vulnerability and lower preparedness during emergencies, such as with the COVID-19 pandemic. Implications of health policies are suggested to improve strategies of crisis management.

## 1. Introduction

Since early 2020, the COVID-19 pandemic has exerted detrimental effects on global healthcare systems, economies, and societies [1]–[7]. Negative impacts have stemmed from a multitude of social, economic, and environmental factors, resulting in a significant increase in infections and fatalities, as well as adverse economic and social consequences in countries [8]–[18]. In the presence of a pandemic crisis, such as COVID-19, one of the main problems is the improvement of health systems and preparedness of countries to address the main challenges posed by pandemics, such as: increased demand for medical care [19], shortages of medical resources [20]–[22], and overburdened healthcare staff [23]–[26]. The preparedness of health systems to cope with the COVID-19 pandemic crisis has been studied from many perspectives:

- **Healthcare Infrastructure**: Many studies analyze the adequacy and capacity of healthcare infrastructure, including hospitals, medical clinics, and testing facilities, to handle the influx of COVID-19 cases [27], [28]. Some studies suggest that countries with better healthcare capacity and enhanced access to medical equipment are better positioned for crisis management of pandemics, such as COVID-19 [13]–[15], [29]–[31].
- **Health Workforce**: Research also focuses on the preparedness of the health workforce to respond to the pandemic crisis, including healthcare professionals, such as doctors, nurses, and allied health workers. The preparedness of human resources in health systems analyzes optimal staffing levels, effective training, improved occupational safety, and workforce resilience [32]–[34].
- **Disease Surveillance and Testing**: Scholars also analyze the effectiveness of disease surveillance systems and testing strategies of countries in detecting and monitoring COVID-19 cases. This aspect includes strategies to improve the availability and accessibility of testing kits, laboratory capacity, effective tracing systems, and the use of digital technologies for surveillance of infected people [35],[36].
- **Health Policy and Governance**: Other studies explore the design of health policy and governance systems in shaping effective preparedness and responses of crisis management. These studies analyze public health interventions (lockdowns, vaccination programs, etc.), emergency response plans based on reduction, readiness, response, and recovery, coordination mechanisms between different institutions, and finally appropriate regulatory frameworks (staying at home, wearing high-quality masks, etc.) [37]–[40].

Some studies focused on economic variables and on preparedness of health systems for a pandemic crisis. Marginean and Orastean [41] examined, during COVID-19 crisis in the European Union, the relationship between health financing and pandemic preparedness. They found that high health spenders performed better in facing the COVID-19 crisis and that high financing increases performance and resilience of health systems [42]. Ovsiannikova [43] found that higher health expenditure reduced COVID-19 mortality in low-income countries but the effect of health expenditure on mortality was insignificant in middle- and high-income countries. Instead, Oshinubi et al. [44] showed that countries with high health expenditure had improved public health strategy to face the second wave of the COVID-19 pandemic. To put it differently, a well-funded healthcare system with adequate economic resources supports pandemic preparedness and is better equipped to manage challenges posed by a pandemic or other health emergency [45], [46]. In short, an adequate financing system ensures that health facilities have the necessary economic and human resources to support strategies of crisis management [29].

Countries often allocate more economic resources to healthcare systems because they have stronger economies, higher economic capacity (based on the gross domestic product), higher levels of disposable income, and larger tax revenues, such that rich countries can invest and spend more resources in healthcare infrastructure, medical technology, and services [47],[48]. Demographic factors also play a vital role to structure health systems. For example, countries with aging populations require more healthcare services and have higher healthcare costs [49]. Instead, countries with high levels of public debt have budget constraints that limit their ability to allocate resources to healthcare systems. In particular, countries with high public debt levels apply economic policies based on fiscal austerity measures, including spending cuts or tax increases, which could negatively impact the funding for healthcare systems [50]–[52]. In fact, high levels of public debt have negative effects on economic systems [53] and can decrease a government's ability to respond to emergencies and social problems [54],[55]. Studies show that high levels of public debt can reduce government expenditure in the health and education sector [56],[57]. In particular, economic policies of debt reduction often decrease health expenditure, a main item of the public budget, and affect the effectiveness of overall health systems [58]. As a consequence, high-debt economies are more vulnerable to crises [59] because debt servicing costs can lead to slower economic growth [53].

In this context, our study had the goal to investigate how high levels of public debt affect health systems and preparedness in European countries to face the COVID-19 pandemic crisis. In particular, the study here aimed to address the following research question:

How do higher levels of public debt affect, associated with other factors, the preparedness of health systems to face a pandemic crisis similar to COVID-19?

**Figure 1.**
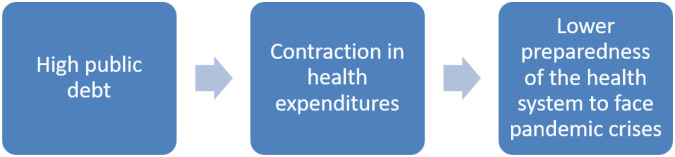
Consequential relation from high public debt to high vulnerability in nations to face health emergencies.

The working hypothesis is that high public debt and the related repayment policies can reduce health expenditures, which lead to a lower preparedness of the health system to manage crises similar to the COVID-19 pandemic, generating a higher fatality rate in society. Figure 1 shows this logical relation.

In short, our research explored, for the first time, the relationship between levels of public debt, healthcare expenditures, and the fatality rates caused by the COVID-19 pandemic in various European countries. A proxy of health system preparedness to face pandemics is the case fatality rate (CFR)–the ratio of COVID-19 deaths to the total number of individuals diagnosed with this novel infectious disease during a specified timeframe [60],[61]. CFR is a preferable indicator to the mortality rate, which is given by the measure of frequency of deaths in a defined population in order to assess health performance of nations during a pandemic crisis [60]. In particular, this study endeavored to explain whether and how high levels of public debt affect the vulnerability of health systems to face an unforeseen crisis, such as the COVID-19 pandemic. The case study of European nations was because they have homogeneous socioeconomic systems and stable structural indicators that facilitate comparative analyses of the relationship between socioeconomic factors and COVID-19 indicators (JHU, 2023). Findings here, based on European countries, can be generalized for broader implications of health policy to improve preparedness and resilience of similar nations in the presence of health emergencies and other crises.

## 2. Research methodology

### 2.1. Sample

Our research is centered on a cohort of 27 European Union (EU) countries characterized by comparable socioeconomic systems, offering a uniform sample conducive to robust statistical analyses. In particular, the sample under study includes the following 27 European countries: Austria, Belgium, Bulgaria, Croatia, Cyprus, Czech Republic, Denmark, Estonia, Finland, France, Germany, Greece, Hungary, Ireland, Italy, Latvia, Lithuania, Luxembourg, Malta, Netherlands, Poland, Portugal, Romania, Slovakia, Slovenia, Spain, and Sweden.

We study the countries in the European Union for four specific reasons:

- **Similar approach to public health**: Many European countries have universal healthcare coverage, ensuring access to essential health services for all citizens. The analysis of the effects of high public debt on health systems can offer valuable implications into maintaining equitable access to care during economic downturns and, in particular, in a period of crisis.
- **Historical context**: Europe has experienced various economic crises and debt challenges throughout its history (i.e., the global financial crisis over 2007–2008 or the sovereign debt crisis from 2009–2013). The analysis of the effects of high public debt on health systems can provide valuable lessons to design effective best practices for crisis management.
- **Policy responses**: European countries have implemented specific economic policies to address problems of high public debt, in particular the austerity measures of Greece, Portugal, and Spain [50]. The effects of these control measures on the performance of health systems are not clear but their study can improve planning and design of future health policies for effective decision-making in crisis management [61].
- **Data availability**: European countries have robust data collection systems by appropriate offices, such as EUROSTAT, which support longitudinal studies for guiding economic policies and health planning of policymakers. This complete dataset facilitates rigorous empirical analyses to explain underlying relationships between public debt, socioeconomic variables, and health systems in the presence of a pandemic crisis and other emergencies.

### 2.2. Variables

In this study, we examined factors related to structural indicators of the economic and health system in European countries during specific years (2009 and 2019) to assess the level and change before the COVID-19 pandemic crisis (starting in February 2020 in Europe) and their relationship with the case fatality ratio of COVID-19 in 2020, at beginning of pandemic crisis, when effective drugs and therapeutic treatments were lacking. Table 1 shows the variables under study here.

**Table 1.** Variables and sources.

Variable and source	Description
Total healthcare expenditures per capita in current US$, 2009 and 2019 WHO (2023)	Per capita total expenditure on health, expressed in current US$.
General government gross debt, annual percentage of gross domestic product (GDP) in 2009 and 2019, Eurostat (2023)	The Treaty on the Functioning of the European Union defines this indicator as the ratio of government debt outstanding at the end of the year to gross domestic product at current market prices. For this calculation, government debt is defined as the total consolidated gross debt at nominal value in the following categories of government liabilities (as defined in ESA 2010): currency and deposits, debt securities and loans. The general government sector comprises the subsectors of central government, state government, local government, and social security funds. For further methodological guidance and interpretation, please refer to the Eurostat *Manual on Government Deficit and Debt*.
General government deficit/surplus as a share of GDP, 2009 and 2019, Eurostat (2024)	Net lending/net borrowing as a share of gross domestic product.
Case fatality rate on 30 December 2020, JHU (2023)	The number of deaths in COVID-19 cases divided by the total number of people infected by COVID-19

### 2.3. Working hypothesis

**Figure 2.**
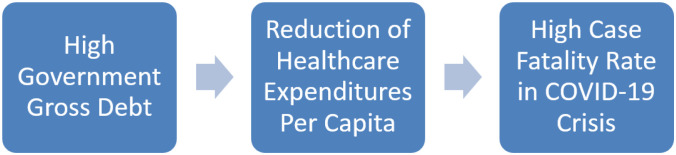
Relationship of high public debt with negative impact on health systems to a high case fatality rate in the presence of the COVID-19 pandemic crisis.

The primary objective of this study was to assess whether statistical evidence supports the working hypothesis that the levels of the COVID-19 fatality rate in European countries can be explained by the level of the general government gross debt (expressed as a percentage of GDP), which seems to have a negative impact on the structure and operation of healthcare systems. Figure 2 shows the logical relationship under study here.

### 2.4. Study design

The variables in Table 1 were examined with descriptive statistics based on arithmetic mean, standard deviation, skewness, and kurtosis. After that, the average COVID-19 fatality rate in the year 2020, the inception year of the COVID-19 pandemic crisis, was employed to categorize the sample of European country into two groups:

- Group 1 - Countries with *lower* COVID-19 fatality rates in 2020 than the sample arithmetic mean: Austria, Croatia, Cyprus, Czech Republic, Denmark, Estonia, Finland, Germany, Latvia, Lithuania, Luxembourg, Malta, Netherlands, Portugal, Slovakia, and Sweden
- Group 2 - Countries with *higher* COVID-19 fatality rates in 2020 than the sample arithmetic mean: Belgium, Bulgaria, France, Greece, Hungary, Ireland, Italy, Poland, Romania, Slovenia, and Spain

The year 2009 is the starting point of current analysis because global financial and economic crisis in 2009 has generated consequential interventions of political economy and public finance to control debt in countries where the national burden was high, such as Italy, Greece, etc.

First, both the arithmetic mean and the change from 2009 to 2019 (ten years) of general government gross debt, of general government deficit/surplus, and of expenditures in health systems are calculated considering countries in groups 1 and 2 to assess the evolution of public debt, government deficit/surplus, and health expenditures before the emergence of the COVID-19 pandemic crisis.

Second, the rate of change for variable *x* is given by:

$$\Delta change\;of\;variable\;x=\frac{\left(x\;in\;2019-x\;in\;2009\right)}{x\;in\;2009}$$

After that, the arithmetic mean of this change (Δ) in groups 1 and 2 is calculated to assess significant differences by using the independent samples *t*-test—i.e., comparing the means to determine whether there is statistical evidence that associated population means are also significantly different. Levene's test was used to check the assumption of homogeneity of variance (i.e., that both groups have the same variance), whereas the hypotheses used for the independent samples *t*-test are:

H_0_: µ1 = µ2, the two-population means of groups 1 and 2 are equal

H_1_: µ1 ≠ µ2, the two-population means of groups 1 and 2 are not equal

Finally, we investigate the relations among general government gross debt (as a percentage of GDP), healthcare expenditures, and COVID-19 case fatality rates in European countries using the two-stage least-squares regression, where an instrumental variable that exhibits no correlation with error terms is employed to calculate estimated values for the predictor (first stage), which is subsequently used to establish a linear regression model for the dependent variable (second stage). Since these computed values rely on variables that are uncorrelated to the errors, the outcomes generated by the two-stage least-squares model are considered optimal results, as explained by Angrist and Krueger (2001).

The two-stage least-squares method is based on the following variables and equations:

- Dependent variable: case fatality rate in 2020
- Explanatory variable (predictor): change of healthcare expenditures per capita US$ from 2009 to 2019
- Instrumental variable: general government gross debt, percentage of GDP in 2009

The two-stage least-squares model is given by following equations:

*Stage 1:*

y_i_ = α + β_1_ x_i_ + u_i_      [1]

y_i_ = change of healthcare expenditures per capita from 2009 to 2019

α = constant

β_1_ = coefficient of regression

x_i_ = general government gross debt, percentage of GDP in 2009

u_i_ = error term

i (subscript) = countries

*Stage 2:*

f _i_ = κ + β_2_ fit y_i_ + ε_i_      [2]

f _i_ = COVID-19 case fatality rate in 2020

κ = constant

β_2_ = coefficient of regression

fit y _i_ = fit for change of healthcare expenditures per capita from 2009 to 2019 with model [1]

ε_i_ = error term

i (subscript)= countries

In equations of the stages 1 and 2, the constant is the value of the dependent variable when the associated predictor or independent variable is equal to zero, whereas the coefficient of regression describes the relationship between a predictor variable and a response variable.

Statistical analyses and the estimation of the two-stage least-squares model that determines the unknown parameters are performed with the IBM SPSS Statistics 26 ®.

## 3. Results

First of all, the arithmetic mean (M) of the case fatality rate on 30 December 2020, in the first year of the COVID-19 pandemic crisis, is M = 1.98% (standard deviation (SD) = 0.86%). This average mean is used to categorize European countries into two groups:

- Countries with a lower COVID-19 fatality rate in 2020 than the sample arithmetic mean, average value is: Mgroup1 = 1.40%
- Countries with a higher COVID-19 fatality rate in 2020 than the sample arithmetic mean, average value within the group is: Mgroup2 = 2.83%

Table 2 shows the arithmetic mean of variables and the rate of change in the two groups just mentioned. Statistical significance of the differences in arithmetic mean between groups 1 and 2 is analyzed by the independent samples *t*-test (and Levene's test). Table 2 reveals that COVID-19 fatality in group 1 was lower in 2020 (1.40%) than group 2 (2.83%).

Group 1, with a lower COVID-19 fatality rate, has, in the years 2009 and 2019, higher levels of health expenditure per capita (>$3100 per capita). From 2009 to 2019, this group 1 has a rate of growth of health expenditure per capita of 0.19.

Instead, countries with a higher COVID-19 fatality rate in 2020 had, in 2009 and 2019, levels of health expenditure per capita lower than the previous group 1 (about $2530 in 2009 and $2600 in 2019). Moreover, group 2 has a lower rate of growth of health expenditure per capita from 2009 to 2019 given by 0.09.

If we consider government gross debt as a percent of GDP, the results of Table 2 reveal that group 1 is lower both in 2009 (50.79%) and 2019 (46.80%) than group 2, which had 81.49% in 2009 and 67.22% in 2019. In addition, group 1 has, from 2009 to 2019, a lower growth of government gross debt (% of GDP) given by 0.12 compared to group 2 that has experienced a high growth in government gross debt (% of GDP) of 0.29, generating a high burden for socioeconomic systems and public finance that has generated negative effects on health expenditures and overall health systems. Average general government deficit/surplus (% of GDP) shows that in 2009, countries with lower COVID-19 fatality were negative but lower than countries with a higher COVID-19 fatality rate (−4.99 versus −8.16), with a similar trend in 2019 with an average value of 0.67 (in countries with lower COVID-19 fatality) versus −1.14 (in countries with higher COVID-19 fatality).

**Table 2.** Descriptive statistics categorized per groups.

	Countries with LOWER COVID-19 Fatality in 2020 (Group 1)		Countries with HIGHER COVID-19 Fatality in 2020 (Group 2)	
Variables	Mean	Std. Deviation	Mean	Std. Deviation
COVID-19 Fatality 2020 (%)	1.40	0.44	2.83	0.54
Healthcare Exp Per Capita $ 2009	$3119.79	$2192.71	$2609.13	$1828.01
Healthcare Exp Per Capita $ 2019	$3376.29	$2014.03	$2530.77	$1749.05
Δ Healthcare Exp Per Capita $ 2009–2019	0.19	0.30	0.09	0.31
Government gross debt, % of GDP 2009	46.79	22.21	67.22	37.35
Government gross debt, % of GDP 2019	50.93	27.43	81.51	46.61
General government deficit/surplus, % of GDP 2009	−4.99	3.06	−8.16	3.80
General government deficit/surplus, % of GDP 2019	0.67	1.28	−1.14	2.01
Δ Government gross debt, % of GDP 2009–2019	0.12	0.31	0.29	0.38
Δ General government deficit/surplus, % of GDP 2009–2019	−5.66	2.77	−7.03	4.41

Note: Δ = the rate of change from 2009 to 2019 to assess the dynamics of health expenditures per capita, government gross debt, and government deficit/surplus before the emergence of the COVID-19 pandemic crisis.

Table 3 presents the results of the independent samples *t*-test, which compares the means of groups 1 and 2 to determine whether the associated population means are significantly different. Since the *p*-value is higher than significance level α = 0.05, we can reject the null hypothesis of similarity of arithmetic means between groups 1 and 2, except for government deficit/surplus (% of GDP) in 2009 and 2019, and the COVID-19 fatality rate in 2020. Table 4 presents the results of the regression analysis using the 2SLS method. In the second stage of the model, the dependent variable is the COVID-19 fatality rate in 2020 for European countries, while the explanatory variable is the change in healthcare expenditure per capita from 2009 to 2019, as determined in the first stage. The findings clearly indicate that when countries experience a 1% increase in healthcare expenditure per capita over the period 2009–2019 (predicted values, accounting for government gross debt as a percentage of GDP in 2009 in the first stage), it leads to a 2.63% reduction in the COVID-19 fatality rate. The R^2^ coefficient of determination explains approximately 25% of the variance in the data. Although the R^2^ value is not particularly high in the model, the *F* value is statistically significant (*p*-value < 0.01), indicating that the independent variable reliably predicts the dependent variable, namely the reduction in the COVID-19 fatality rate.

**Table 3.** Independent Samples Test based on average mean of change in variables from 2009 to 2019 for European countries of group 1 (Countries with LOWER COVID-19 Fatality in 2020) and group 2 (Countries with HIGHER COVID-19 Fatality in 2020).

	Equal Variances	Levene's Test for Equality of Variances		*t*-test for Equality of Means		
		*F*	Sig.	*t-test*	Degrees of freedom	Sig. (2-tailed)
Healthcare Exp per Capita $ 2009	assumed	1.358	0.255	0.635	25	0.531
	not assumed			0.657	23.947	0.517
Healthcare Exp per Capita $ 2019	assumed	2.095	0.16	1.129	25	0.270
	not assumed			1.16	23.515	0.258
Δ Healthcare Exp per Capita $ 2009–2019	assumed	0.214	0.648	0.828	25	0.416
	not assumed			0.826	21.541	0.418
Government gross debt, % of GDP 2009	assumed	4.609	0.042	−1.784	25	0.087
	not assumed			−1.626	14.865	0.125
Government gross debt, % of GDP 2019	assumed	3.460	0.075	−2.163	25	0.040
	not assumed			−1.966	14.702	0.068
Δ Government gross debt, % of GDP 2009–2019	assumed	0.64	0.431	−1.275	25	0.214
	not assumed			−1.23	18.852	0.234
Government deficit/surplus, % GDP, 2009	assumed	0.851	0.365	2.401	25	0.024
	not assumed			2.304	18.469	0.033
Government deficit/surplus, % GDP, 2019	assumed	4.594	0.042	2.863	25	0.008
	not assumed			2.639	15.569	0.018
Δ Government deficit/surplus, % GDP, 2019–2009	assumed	1.298	0.265	−0.995	25	0.329
	not assumed			−0.915	15.402	0.374
COVID-19 fatality rate in 2020	assumed	0.698	0.411	−7.518	25	0.001
	not assumed			−7.245	18.775	0.001

**Table 4.** Parametric estimates.

	Constant	Coefficient of regression *β*	Standardized coefficient of regression *β*	R^2^	*F*
*Stage 1*					
Change of healthcare expenditures per capita US$ in 2009–2019 (1)	0.449***	−0.005**	−0.540	0.29	10.27**
*Stage 2*					
COVID-19 case fatality rate 2020 (2)	2.383***	−2.626**	−0.502	0.25	8.41**

Note: *** p < 0.001; ** p < 0.01. (1) Explanatory variable: General government gross debt, percentage of GDP in 2009; (2) Explanatory variable: Fit for change of healthcare expenditures per capita from 2009 to 2019 with model of stage 1. R^2^ is the coefficient of determination. F is the ratio of the variance explained by the model to the unexplained variance.

**Figure 3.**
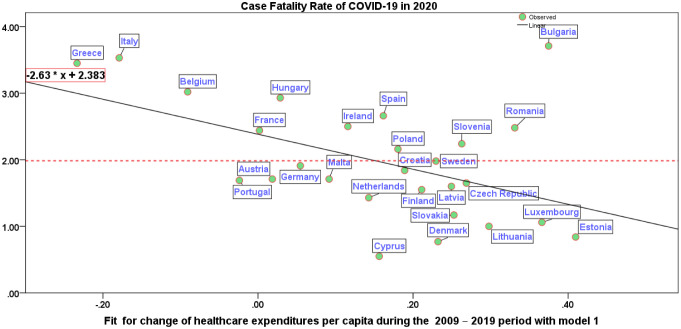
Regression line of the COVID-19 fatality rate in 2020 on the fit for change of healthcare expenditures per capita during the 2009 – 2019 period with a model of stage 1. Note: The area of vulnerability has CFR > 2.00, resilient countries have CFR < 2.00; CFR = case fatality rate. R^2^ = 0.25, *F*-test = 8.41 (*p*-value = 0.008). Countries with *higher* COVID-19 fatality rates (above the line of 2.00, which are vulnerable countries to a pandemic crisis) are: Belgium, Bulgaria, France, Greece, Hungary, Ireland, Italy, Poland, Romania, Slovenia and Spain. Countries with *lower* COVID-19 fatality rates (below the line 2.00, which are resilient countries to a pandemic crisis) are: Austria, Croatia, Cyprus, Czech Republic, Denmark, Estonia, Finland, Germany, Latvia, Lithuania, Luxembourg, Malta, Netherlands, Portugal, Slovakia, and Sweden.

Figure 3 illustrates the estimated relationship and average line on the *y*-axis. Figure 3 also shows countries above the horizontal line (with an average value of 2.0), which have a higher vulnerability in coping with the COVID-19 pandemic crisis, experiencing a high fatality rate, such as Belgium, Hungary, Spain, Poland, Slovenia, Romania, and notably Greece and Italy. Conversely, countries located below the average line in Figure 3 show a greater resilience in healthcare systems and lower case fatality rates in 2020 in the presence of the COVID-19 pandemic crisis [13],[62].

## 4. Discussion

Results show that countries with a lower COVID-19 fatality rate have higher levels of healthcare expenditure per capita (including physicians, nurses, hospital beds, preventive care, and curative acute care). Conversely, European nations experiencing higher COVID-19 fatality rates exhibit lower healthcare expenditure per capita, which was approximately $2600 in 2019. Regression analysis indicates that countries having a 1% increase in the change of healthcare expenditure per capita from 2009 to 2019 (predicted values based on government gross debt as a percentage of GDP in 2009 in the initial stage) have a notable 2.63% decrease in their COVID-19 fatality rates.

### 4.1. Explanation of results

Expenditures and investments in health systems have a vital role to support the well-being of people and also to be prepared to face pandemic crises and general health emergencies [13],[63],[14]. Countries that allocate a greater amount of resources to the healthcare sector generally have more prepared healthcare systems to deal with various types of crises, such as epidemic outbreaks, natural disasters, conflicts, or even long-term challenges such as chronic diseases [64]. Nations that prioritize the allocation of substantial economic resources toward the healthcare sector are able to achieve higher levels of preparedness and resilience in the presence of unforeseen health emergencies, since their healthcare systems are bolstered by solid infrastructure, adequate medical supplies and equipment, and a well-trained healthcare workforce [41]. This enhanced capacity in nations enables earlier and more precise diagnoses of diseases, more effective treatment options, and improved crisis management in the presence of health emergencies [65].

Our data also suggests that high public debt in European countries leads to lower health expenditure per capita. In the period 2009–2019, countries with a higher fatality rate had a great growth in public debt (0.29% of GDP). This dynamic has led to a reduction in total healthcare expenses per capita. In the same period, countries with a lower COVID-19 fatality rate, despite a smaller growth in public debt (0.12 of GDP), recorded a significant increase in health spending, amounting to 0.19% of GDP. High levels of public debt are therefore associated with a lower level of healthcare expenditure and preparedness of health systems to deal with the COVID-19 pandemic crisis, generating a high fatality rate. In short, countries with a higher level of public debt have experienced a greater vulnerability to face the COVID-19 pandemic crisis. Greece and Italy, for example, had high level of government debt as a percent of the GDP that has limited health expenditure, deteriorated the overall health system, and affected consequential preparedness to face and recover from health emergencies [62]. According to Theodoropoulou [58], the pandemic hit hard in Greece because the healthcare system was one of the main targets of public spending cuts under the economic adjustment programs of the 2010s. The systemic vulnerability, associated with high public debt in some countries, is often due to interventions of political economy based on austerity measures (e.g., the European Stability and Growth Pact, SGP) that reduce the burden of high government debt with cuts to public expenditure. SGP is a set of rules designed to make sure countries in the European Union (EU) have good control of their finances, and countries with the goal of reducing high public debt apply SGP rules [54],[58]. Nickel et al. [66] found that the financial crisis of 2008–2009 has left European economies with a sizeable public debt stock. They suggest that major debt reductions were mainly driven by decisive and lasting fiscal consolidation efforts focused on reducing government expenditure through cuts in social benefits, health services, and public wages. In general, the reduction in social and health spending is often triggered by economic policies to combat high public debt. Köhler-Töglhofer and Zagler [67] showed that a high debt burden, after a fiscal expansion, will constrain policy intervention in the future and a high level of public debt will be a drag on financial markets in the entire union. Reductions in government expenditures can lead to a dampening of debt dynamics across all fiscal policy regimes, such that expenditure cuts are more important for debt reductions than revenue increases. Iwata and Iiboshi [68] argued that the increased magnitude of fiscal adjustments appears to be the major driving force behind the decline in government spending multipliers rather than debt accumulation itself. Of course, these public policies do not consider the effects of high public debt on the systemic resilience of a nation to face crises. Burriel et al. [59] analyzed economic risks in regimes of high public debt associated with the 2009 global financial and economic crisis and the more severe COVID-19 pandemic crisis. These scholars suggested that high-debt economies have more reduction of output in a crisis and have less scope for counter-cyclical fiscal policy leading to both high debt levels and higher socioeconomic vulnerabilities. ECB [69] showed that high government debt generates economies that are less resilient to shocks. In particular, high public debt exerts adverse pressure on the economic system through multiple channels, such as lower real growth or inflation shocks that increase the real burden of debt, with larger fiscal costs if the initial level of debt is high. De Soyres et al. [70] showed that the impact of an unanticipated increase in public debt on the real GDP level is generally negative, particularly for countries that have a high initial debt level or a rising debt trajectory over the five preceding years. Heimberger [71] suggested that a 10 percentage points increase in public debt-to-GDP is associated with a decline in annual growth rates by 0.14 percentage points. Panizza and Presbitero [72] also asserted that there was no empirical evidence to support the results that public debt has a causal impact on economic growth. This finding holds significant importance because the negative correlation between public debt and growth has been used to justify specific public policies assuming that debt inherently hampers economic growth. Furthermore, Fan et al. [73] showed that the dynamics of debt and economic growth vary significantly according to the type of crisis. In this context, Fan et al. [73] reported a rapid increase in public debt across economies during 2020–2021, primarily because of the global COVID-19 pandemic crisis. Georgantas et al. [74] maintained that fiscal and spending adjustments made during recessions, especially in periods of tight monetary conditions and when the debt ratio exceeds 80%, tend to be counterproductive. In contrast, fiscal consolidation efforts initiated during economic expansions, particularly in low-debt countries, and with accommodating monetary conditions of open economies, can result in a more substantial reduction in the debt ratio. In short, in the presence of the potentially devastating effects of various types of crises that can happen anytime, such as the COVID-19 pandemic crisis, it is important to maintain a country's ability to respond quickly with a resilient system, and this strategy cannot be done with high and rising public debt that deteriorates socioeconomic systems with cuts (to health and other sectors), generating negative effects in society, such as high fatality. In general, studies by CBO [75] argued that the main consequences of high public debts are lower national savings and income, higher interest payments, large tax hikes, and spending cuts. These effects generate a decreased resilience and ability to respond to environmental risks and crises in countries [76]. Lessons learned from the study here are that recessions and crises could have larger negative effects on socioeconomic systems and people's well-being in countries with high public debt because of previous general cuts to expenses, investments, and other interventions in health and other systems. McKee et al. [77] argued that many governments in Europe, either of their own volition or at the behest of the international financial institutions, have adopted stringent austerity policies in response to high levels of public debt. However, austerity in Europe has not been only an economic failure, but also a failure of the health sector, with cuts in health budgets [50] that increased the number of people unable to receive appropriate treatments, such as during the COVID-19 pandemic crisis when medical ventilators and intensive care units were insufficient to treat infected individuals, generating higher numbers of deaths.

Hence, the public policy of restriction to control high public debts has increased the systemic vulnerability and reduced resilience of health systems in many European countries to face emergencies.

### 4.2. General deduction of the analysis of findings

The main findings of our study can be summarized in the following two statements:

- High public debt leads to lower health expenditure and reduces health system preparedness for crisis management.
- European countries with lower public debt and higher health expenditures per capita have higher resilience to face pandemic crises, which reduced COVID-19 fatality rates.

## 5. Concluding observations

The Global Burden of Disease [78] group has showed that the COVID-19 pandemic highlighted gaps in disease prevention and treatment between European countries and worldwide. One of the manifold factors of these gaps is the issue of reduced financing in national health systems because of austerity measures for reducing high levels of public debt [79]. During pandemic crises, nations strive to achieve two primary objectives: reducing mortality rates and upholding the stability of their socioeconomic systems, as emphasized by Coccia [13]. The statistical findings here seem to support the working hypothesis outlined in Section 2: *High case fatality rates of COVID-19 can be explained by a high burden of public debt in socioeconomic system, which constrains healthcare expenditures across European countries*. In particular, the findings here suggest that countries with a high average level of government debt as a percent of the GDP over 2009–2019 have lower health spending per capita because of constraints from austerity economic policies [79] that have generated negative effects in society with high levels of COVID-19 case fatality rate. The findings presented here also indicate that a 1% increase in the change of healthcare expenditures per capita from 2009 to 2019 is associated with an approximately 2.63% reduction in the COVID-19 fatality rate. In short, austerity policies to keep debt ratios at prudent levels and avoid further sovereign debt crises and financial shocks lead to general cuts of expenses, including in the health sector, which expose countries to vulnerabilities when facing pandemic crises (and other emergencies), reinforcing detrimental effects on national output and welfare in society.

### 5.1. Health policy implications to face emergencies

The results of our study allow us to propose some implications for health planning in countries to face future crises. In particular, to gain a high level of resilience in the presence of crises, best practices of health policy should be based on:

**a. Prioritizing preparedness by avoiding broad budget cuts and reinforcing health expenditures.** Coccia and Benati [80] showed that public health systems play a basic role during health emergencies, such as the COVID-19 pandemic crisis, but governments invest relatively little in public health in many countries. Nations must prioritize preparedness to face pandemic crises by investing in the health sector to support robust public health infrastructures and effective disease surveillance systems and apply responsive health policy measures to prevent and/or control outbreaks and improve patient treatments. Consequently, prioritizing investments in healthcare infrastructure or augmenting healthcare expenditures, rather than implementing broad budget cuts based on austerity policies, can lead to a substantial enhancement of health system performance and preparedness to counter pandemics threats during times of crisis within a national and global context. This strategy should be applied also in countries having high levels of public debt. The study here revealed that the relationship between health expenditure and fatality rate of COVID-19 is affected by the level of public debt and spending cuts based on austerity measures to avoid financial crises [79]. However, drastic cutting to health expenditure exposes countries to systemic risks in the presence of pandemics and other crises. Moreover, fiscal austerity reduces both public health and curative expenditures, increasing national vulnerability to emergencies. Lessons from the COVID-19 pandemic crisis show the significance of directing economic resources toward healthcare systems and bolstering public health infrastructure. Coccia [81] argued that to support preparedness for future crises, it is important to foster investments for reducing health inequalities and improving infrastructure and diffusion of medical technologies. The Global Burden of Disease [78] group suggests that there is a unique opportunity to sustain funding for improving vital health functions, including pandemic preparedness with surveillance systems, tracing systems, and effective healthcare infrastructures. In fact, historical patterns of underfunding in the health sector, after the financial crisis of 2009, suggest that deliberate efforts must be done to support health funding directed to improve preparedness for future pandemics and other emergencies [63],[65],[82]–[84]. Sagan et al. [62] maintained that enhancing health system resilience is based on reinforcing health expenditures for all functions of the health sector associated with effective governance, which is the adhesive factor for a systemic resilience in countries. Although a high level of public debt, the long-run strategy should be directed to mitigate cuts in the health sector [79]. In fact, higher health expenditures support health systems resilience that nowadays have expanded the function to aspects of how to minimize exposure to shocks (and manage risks) and to identify timely and effective measures that address more predictable and enduring system strains or stresses for different types of crises (pandemics, conflicts, etc.) [64].

**b. Good governance**. Good governance is crucial to support higher preparedness and resilience efforts during crises [35],[85]. Effective governance ensures efficient allocation of resources and coordination between institutions for timely responses that improve the strategies of crisis management. Higher preparedness and resilience of nations to face pandemic crises have to be supported with higher health expenditures per capita associated with good governance to create efficient public health infrastructures, effective disease surveillance systems, and trained human resources for effective health policy responses, which help to control outbreaks and improve treatment of patients [35],[62],[85],[86]. Effective governance in institutions should be a means to reduce vulnerability during pandemic crises (and other emergencies) and safeguard populations from negative effects [65],[85]. Overall, regardless of the level of high public debt, higher health expenditures have to be supported by good governance in institutions that are basic aspects for timely and effective responses in crisis management.

**c. Promote European Union investment and a systemic approach to face emergencies**. The European Union should increase investment in common surveillance systems, joint procurement initiatives, and targeted funding for a comprehensive and stable system of preparedness for crisis management [87]. These strategies for improving the preparedness and resilience of nations to face future pandemic crises and other emergencies (e.g., conflicts) should be based on a systemic approach, going beyond strengthening health systems and incorporating best practices of good governance in all institutions to reduce negative effects on socioeconomic systems between countries [88]–[90]. A systemic approach is a basic strategy in Europe that has interrelated national systems, of which the health system is just one element. Hence, strategies to increase expenditures and investments in health sectors do not have to be an isolated public policy but they have to be part of broader and systemic multi-sectorial strategy to effectively enhance national resilience of overall countries operating in the European area [91]–[93],[62].

**d. Strategy oriented to new medical technologies**. Technological investments in the healthcare sector, such as mechanical ventilators and other medical technologies, play a pivotal role in bolstering a country's preparedness and resilience in the presence of new and unforeseen infectious diseases of the respiratory tract [15]. In other words, the preparedness of nations for future pandemic crises of airborne diseases should also be directed to investments in new technologies for a modern healthcare system within a framework of effective governance across all institutions. This strategy oriented to medical technologies, also considering new trajectories in digital technology and generative artificial intelligence, is basic for crisis management to support effective treatments when drugs for new viral agents are lacking [14],[82],[86],[94]–[96].

Hence, considering the results of this study, a basic aspect of coping with pandemics is systematic planning, which should consider continuous investments in health sectors to support healthcare infrastructure, equipment, technology, staffing, and training, regardless of the level of public debt.

### 5.2. Limitations and ideas for future research

The analysis of these findings seems to suggest that there is a link between effective health system preparedness to face pandemic crises, higher health expenditures, and a lower level of public debt in countries. It is important to note that these conclusions are preliminary results. The study used public debt and health expenditure data from specific years, which may not reflect longer-term trends or sudden economic changes. In addition, the relationship between public debt, health expenditure, and pandemic preparedness is complex and may involve other variables not considered in this paper. Moreover, the current analysis does not account for all potential confounding factors that can influence the relationship between COVID-19 fatality rates, the level of public debt, and healthcare expenditures. It is imperative to acknowledge the need for further extensive research in this scientific field.

Therefore, it is essential to conduct additional investigations, considering new health system factors and structural indicators, to gain a more comprehensive understanding of the determinants of structural vulnerabilities to face emergencies in countries. The discovery here about negative effects of high public debts in countries for supporting health expenditures has the potential to shed light on the fundamental and structural elements within economic systems that generate economic and institutional weaknesses in specific European nations to cope with pandemic crises (and other emergencies), resulting in elevated fatality rates in society.

To conclude, preparedness and resilience of nations to face crises and reduce negative effects of future potential pandemics (similar to COVID-19) and other emergencies (e.g., conflicts) should be based on systemic economic policies of public debt reduction without decreasing expenses in the sector of public health that play a critical role in crisis management for effective policy responses to maintain the well-being of people and support the operation of socioeconomic systems.

## Use of AI tools declaration

The authors declare they have not used artificial intelligence (AI) tools in the creation of this article.
